# P-818. Unraveling Mortality Drivers in Gram-negative Bloodstream Infections to Guide Antimicrobial Stewardship Efforts

**DOI:** 10.1093/ofid/ofae631.1010

**Published:** 2025-01-29

**Authors:** David S Burgess, Katie B Olney, Donna R Burgess, Sarah Cotner, Jeremy VanHoose, Leah Catherine Turner, Rebecca Bruning

**Affiliations:** University of Kentucky College of Pharmacy, Lexington, Kentucky; University of Kentucky HealthCare, Lexington, Kentucky; UK HealthCare, Lexington, KY; UK HealthCare, Lexington, KY; UK HealthCare, Lexington, KY; University of Kentucky College of Pharmacy, Lexington, Kentucky; Moffitt Cancer Center, Tampa, Florida

## Abstract

**Background:**

Bloodstream infections (BSIs) represent a significant clinical challenge due to their association with substantial morbidity and mortality. This study aimed to identify predictors of mortality in Gram-negative BSIs to guide more effective antimicrobial stewardship strategies.

**Methods:**

Adult inpatients with Gram-negative BSIs from our academic medical institution between July 2022 and August 2023 were included. Data collected included patient demographics, microbiological data, clinical management variables (qPitt, Charlson Comorbidity Index (CCI), ICU admission, length of stay), ID consultation, and clinical outcomes. Logistic regression and CART analysis identified mortality predictors.

**Results:**

In total, 441 predominately Caucasian patients (85.7%), with a mean age 58.5 yrs and 49.4% male, were included. Majority (61.5%) of infections were community-acquired, with 43.1% requiring ICU admission and 43.8% receiving an ID consultation. Overall mortality was 20.0% with the following prevalence and mortality rates: *E. coli* (39.7%, 14.9%)*, K. pneumoniae* (14.1%, 17.7%)*, P. aeruginosa* (9.1%, 35.0%), *S. marcescens* (7.5%, 24.2%), and *E. cloacae* (5.2%, 17.4%). Multivariable regression identified hospital-acquired infection (p < 0.001), qPitt > 2 (p < 0.001), and ICU admission (0.001) as independently associated with increased mortality. Additionally, renal disease (p=0.025), cancer (p = 0.026), and cardiovascular disease (p=0.040) were significant contributors to mortality. CART highlighted ICU admission as the primary driver of mortality, with higher mortality rates compared to non-ICU admissions (32.1% vs 10.8%, p< 0.001). Among ICU patients, a CCI >4 was identified as a secondary driver of mortality, with rates of 14.5% for CCI < 4 and 39.0% for CCI >4, p=0.001. Non-ICU patients with a length of stay < 8 days had lower mortality (3.3% vs 16.6%, p = 0.001). ICU patients received fewer ID consultations compared to non-ICU patients (49.3% vs 36.7%, p =0.007).

**Conclusion:**

This study underscores the impact of Gram-negative BSIs, particularly regarding ICU admission’s role in mortality. Addressing disparities in ID consultations offers opportunities for optimization of patient outcomes through enhanced care coordination.

**Disclosures:**

**Sarah Cotner, PharmD, BCPS**, Gilead Sciences, Inc: Advisor/Consultant

